# In silico evaluation of geometry variations with respect to the thermal spread during coagulation of egg white using bipolar vessel sealing instruments

**DOI:** 10.1186/s12938-016-0238-2

**Published:** 2016-11-02

**Authors:** Jay Wagenpfeil, Christina Schöllig, Volker Mayer, Ronny Feuer, Bernhard Nold, Alexander Neugebauer, Michael Ederer, Ralf Rothmund, Bernhard Krämer, Sara Brucker, Markus Enderle, Oliver Sawodny, Julia Rex

**Affiliations:** 1 Institute for System Dynamics, University of Stuttgart, Waldburgstr. 19, 70563 Stuttgart, Germany; 2 Medical Technology Group, Technische Universität Berlin, Dovestr. 6, 10587 Berlin, Germany; 3 Erbe Elektromedizin GmbH, Waldhörnlestr. 17, 72072 Tübingen, Germany; 4 University Women’s Hospital, Calwerstr. 7, 72076 Tübingen, Germany

**Keywords:** Bipolar vessel sealing, Thermal spread, Mathematical modeling, Finite element analysis, Egg white model

## Abstract

**Background:**

Bipolar vessel sealing is an efficient electrosurgical procedure for the occlusion of blood vessels particularly during minimally invasive surgery. Reliable knowledge of the thermal spread is crucial for a safe application of bipolar vessel sealing instruments when operating close to thermo-sensitive structures, such as nerves. The evolution of the thermal spread over time and space depends on a variety of parameters, such as the biological tissue, the energy applied to the tissue, and the geometry of the vessel sealing instrument. Mathematical modeling has proven useful for the prediction of the thermal spread. It is, thus, a promising tool for the systematic analysis of the influence of geometrical changes on the thermal spread.

**Results:**

We present an experimentally validated in silico study to evaluate the impact of geometry variations on the progression of chicken egg white coagulation and the final shape of coagulated egg white as an approximation of the temporal and spatial evolution of the thermal spread during bipolar vessel sealing. Egg white has similar thermal and electrical properties to human tissue, with the advantage being that the spatial and temporal evolution of the thermal spread can be visually gauged. The simulations were performed using a mathematical model based on the finite element analysis of chicken egg white. The progression of egg white coagulation was predicted for two different peak voltages and various electrode geometries. Starting with two planar electrodes, one electrode was gradually changed to adopt a wedge shape. These changes to the geometry showed a distinct influence on the progression of egg white coagulation in the simulations. The predictions were successfully validated using an experimental setup with two different electrodes representing the extreme geometries.

**Discussion:**

The predicted spatial temperature distributions were experimentally validated for two geometries. Our simulation study shows that the geometry has a pronounced influence on the thermal spread and, thus, is a suitable parameter to reduce thermal damage. The in silico optimization of instrument designs is a suitable tool to accelerate the development of new vessel sealing instruments, with only a few promising designs having to be tested as prototypes.

## Background

**Fig. 1:**
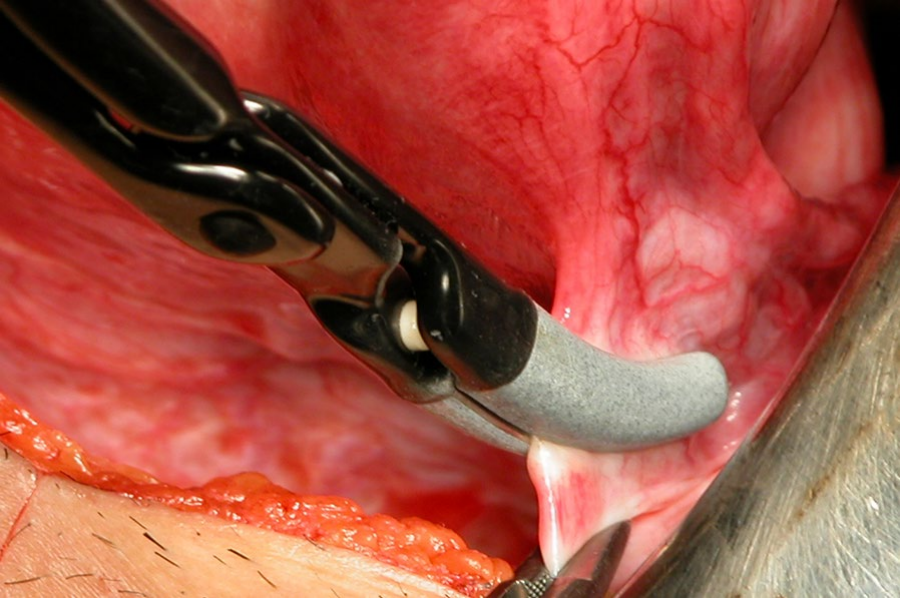
Exemplary application of a bipolar vessel sealing instrument. Vascular occlusion during vaginal hysterectomy using the Erbe BiClamp$^\circledR$ 201 C. Image courtesy of Erbe Elektromedizin GmbH

Bipolar vessel sealing systems are typically composed of an interchangeable thermofusion instrument, consisting of two electrodes on the inside of a forceps and an isolator, as well as a radio-frequency alternating current generator. During radiofrequency-induced thermofusion, the blood vessel is grasped and compressed with the thermofusion instrument (see Fig. 1). An alternating current is applied causing Joule heating of the tissue and, hence, the denaturation of collagen and other proteins that eventually lead to vessel occlusion [1]. Today, thermofusion is a well-established technique in both open and minimally-invasive surgery for the safe and reliable sealing of blood vessels up to 7 mm in diameter [2]. Recent investigations demonstrate that also larger vessels with a diameter between 7 and 12 mm can be successfully sealed using bipolar thermofusion if the compression force is increased [3] or if the vessel is sealed twice [4]. A major advantage of bipolar vessel sealing is the nearly bloodless preparation of tissue structures in a short time without foreign matter remaining in the body. This significantly reduces surgical time and postoperative wound bleeding compared to conventional ligation [5, 6]. Yet, there is still considerable potential for improvement and the process of thermofusion itself has yet to be fully understood. One challenge is the thermal damage of the lateral tissue, especially when operating in the proximity to thermosensitive structures, such as other nearby vessels [7] or, more importantly, nerves, where temperatures of only 43 °C could irreversibly impair the neural tissue [8]. This usually leads to a significantly reduced quality of life, for example, when thermal damage of the nearby neurovascular bundle during radical prostatectomy impairs urinary continence and erectile function [9].

An improved understanding of the parameters influencing the extent of the thermal spread into the lateral tissue is essential for a substantiated assessment and advancement of thermofusion instruments, an understanding that allows for safe usage, also in more delicate surgical procedures. Several studies compared different electrothermal vessel sealing methods and instruments, using both ex vivo  [10, 11] and in vivo models [12–15], and investigated the thermal spread. The thermal damage to the tissue is generally investigated using histomorphological analysis at the end of the thermofusion process. A major disadvantage of this technique is that only the resulting overall damage to the tissue can be captured without consideration of the dynamic distribution of the thermal spread during the process. Information about the temperature distribution over time and space can be partially provided by the use of infrared cameras. Thermal imaging is, however, limited to measuring the surface temperature of the tissue. Sensors were therefore inserted into the tissue in a few studies to measure temperatures below the surface. Due to restricted space, the number of sensors and thus the information provided remains limited.

A mathematical model of the vessel sealing process allows for a more systematic investigation and provides deeper insight into the evolution of the process variables, such as the tissue temperature at any given point in time and space. Most of the reported models use finite elements (FE) for a coupled simulation of the electric field and the tissue’s temperature. A major challenge is the high complexity and the validation of such models, which has thus far significantly limited the application of mathematical models in electrosurgery. Dodde et al. [16] developed a thermo-electric model to study the temperature distribution during bipolar electrosurgery. This model was then extended to account for changes in the specific heat capacity, electrical resistance and water contents of the tissue during the thermofusion process [17]. Gonzalez-Suarez et al. [18] developed another FE model to investigate the thermal spread when using a bipolar instrument with internally cooled electrodes. In a previous work [19] we established an egg-white model for the experimental investigation of the thermal spread caused by bipolar vessel sealing devices. This experimental model was accompanied by a validated mathematical model of the coupled heat transfer and electrostatic partial differential equations (PDEs). The mathematical model allows for the detailed study of the thermal spread’s progression and local inhomogeneities of the spatial temperature distribution. Since egg-white has thermal and electrical properties very similar to those of human or animal tissue with high water contents [20, 21], it is a suitable replacement for tissue in many cases, with the benefit being that egg white is much more available than animal tissue. The main benefit of using egg white is the fact that the denaturation process—which occurs between 57 and 63 °C—can be visually observed. This allows for the verification and validation of the mathematical model’s transient behavior, particularly with respect to the formation of hotspots and the evolution of the thermal spread.

These studies demonstrate that mathematical modeling is a valid tool for analyzing the influence of process parameters on the temperature distribution before experimental testing. An important parameter is the design and geometry of the bipolar instrument’s forceps. Improved forceps designs have the potential of significantly reducing thermal damage [22]. Zhao et al. [23] compared a concave–convex electrode geometry with a conventional smooth electrode during colonic anastomoses. They found that the concave–convex geometry positively influenced the thermal diffusion and could possibly reduce the risk of thermal necrosis. Rothmund et al. [15] found that the use of instruments with large electrodes and correspondingly large heat capacity may have an adverse influence on the thermal damage under certain circumstances. Several other studies, e.g., [11, 16, 24], have shown that thermal damage can differ significantly between vessel sealing instruments. A systematic optimization of the instrument geometry remains, however, difficult. So far, the largest challenge is there is no way to assess the effect of geometry changes a priori. Instead, it has to be evaluated in experimental testing with a sufficiently high sample size. This is a time-consuming and cost-intensive process and, due to the high variability of biological tissue [25], it is often difficult to deduct a clear relationship. Here, a validated mathematical model can unfold its full potential.

The aim of this study is to investigate variations of the electrode geometry with respect to the spatial and temporal evolution of the thermal spread during the coagulation of egg white. We use our previously developed mathematical model [19] to evaluate the influence of geometry variations in silico. The thermal spread predicted by the mathematical model is experimentally validated for two geometries. The proposed combination of in silico analysis of parameter variations and few experimental validations of distinct parameter combinations has the potential to drastically reduce the number of prototypes required and thus accelerate the development process.

In the following, we will illustrate how a mathematical process model can be used to predict the coagulation of egg white for different instrument geometries. The results will be evaluated with particular focus on the formation of hotspots and the spatial temperature distribution, i.e. the shape of the coagulated egg white in the predictions. The paper is organized as follows. In "Methods" section, the mathematical modeling approach is briefly reviewed and the simulation study as well as the corresponding experimental setup is described. The results of the simulation study and the validation experiments are presented in "Results" section  and discussed in "Discussion" section. A properly verified model is the basic requirement for obtaining meaningful predictions. The simulations in [19] were obtained using slightly differing values of the electrical conductivity of egg white, which resulted in small errors with respect to the temporal dynamics. These errors did not affect the general statement of [19], but with regard to the goal of the study at hand, proper verification results are given in Appendix 2.

## Methods

### Mathematical model

The radiofrequency-induced electrical heating of chicken egg white can be mathematically described by the partial differential equation (PDE) system

1 $$\begin{aligned} \rho c \frac{{\partial T}}{{\partial t}}&= \nabla \left[ k \nabla T \right] + q_\mathrm {el} \end{aligned}$$

2 $$\begin{aligned} 0&= \nabla \left[ \sigma \nabla V \right] \end{aligned}$$

with proper initial and boundary conditions. This thermal-electrical system is comprised of the heat equation (1), and also the Laplace equation (2), since electromagnetic coupling can be typically neglected during RF-induced electric heating [26]. The two PDEs are coupled through the temperature dependent electrical conductivity $\sigma = \sigma (T)$ and the heat flux due to Joule heating

3 $$\begin{aligned} q_\mathrm {el}= \sigma {(\nabla V)}^\mathrm {T} \nabla V. \end{aligned}$$

The state variables are the temperature $T = T(t,\xi )$ and the electric potential $V = V(t,\xi )$. The independent variables are the time *t* and the spatial coordinates $\xi$. The density $\rho$, the heat capacity *c*, and the thermal conductivity *k* are assumed to be constant in the relevant temperature range. Please refer to Table 1 for the parameter values used in the simulations. Refer to Appendix 1 for a detailed description of crucial parameter values and their derivation, particularly the temperature dependent conductivity of egg white $\sigma (T)$ which has a distinct influence on the coagulation process.

**Table 1:** Material properties used in the simulations

Property	Egg white	Electrode	Isolator
Density $\rho \; ({\rm kg}/{{\rm m}^3})$	1030	8000	1030
Specific heat capacity $c \; ({\rm kJ}/{{\rm kg}\,{\rm K}})$	3.550	500	2200
Thermal conductivity $k \; ({\rm W}/{{\rm m}\,{\rm K}})$	0.55	15	0.25
Electrical conductivity $\sigma \; ({\rm S}{\rm m})$	0.74–1.9	1.33 × 10^6^	1.0 × 10^−12^

Please refer to the Appendix for a detailed derivation of the temperature dependent electrical conductivity of egg white

### Simulation study and experimental setup

For the simulation results presented in this paper, the PDE system was discretized via the Finite Element Method (FEM) using the software ANSYS 15.0. Starting from a typical instrument geometry with two planar electrodes, one of the two electrodes increasingly assumed a wedge shape. The planar electrode had a conductive area of 5.7 by 20 mm and a thickness of 4.7 mm. The electrodes were covered with a  0.15 mm thick isolating material on the non-conducting surfaces. Five different heights of the wedge were investigated, namely 0, 0.1, 0.5, 1.0 and 2.85 mm, corresponding to wedge angles of 180°, 176°, 160°, 141° and 90° respectively. The wedge was added to one of the two electrodes. Zero wedge height, respectively a wedge angle of 180°, is equivalent to a planar electrode. The distance between the tip of the wedge and the opposing planar electrode was kept at 1 mm independent of the wedge height. In our previous work we saw that the shape of the coagulated egg white distinctly differed depending on the applied voltage. Therefore, two generator settings with peak voltages of 35 and 165 V_p_, respectively, were selected for the simulation study.

**Fig. 2:**
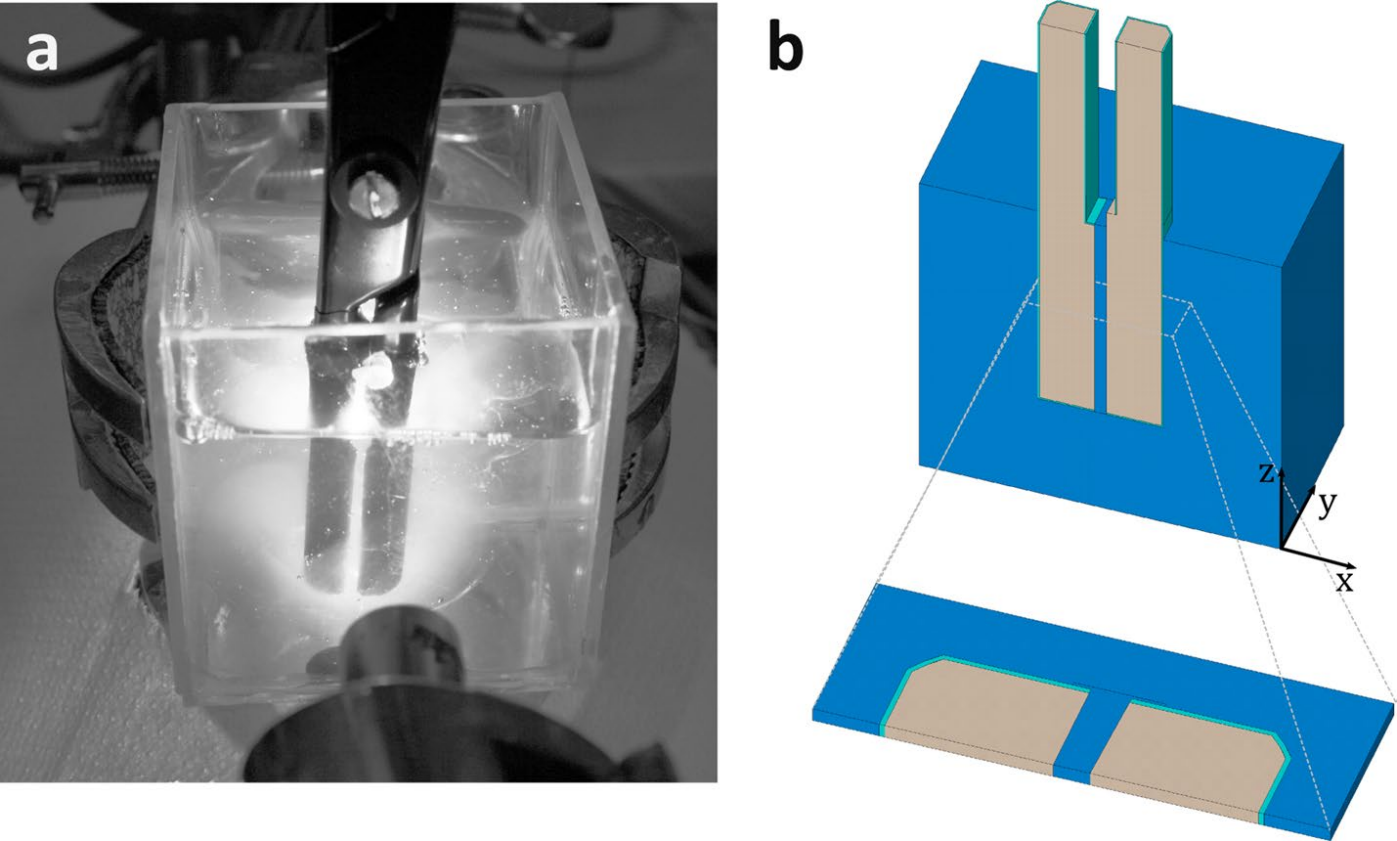
Egg white coagulation with a vessel sealing instrument. **a** The setup of the experiment that was used for model validation with prototype instrument A. **b** Corresponding FE model of the whole instrument with surrounding egg white and central cutout of the instrument

Validation experiments with two prototype vessel sealing instruments supported the model’s predictions. The first instrument, A, had two planar electrodes while the second instrument, B, has one planar electrode and one wedge-shaped electrode with an angle of 90°. The instruments were vertically immersed into a beaker filled with egg white until the electrodes were completely submerged (see Fig. 2a). Both instruments were adjusted so that the electrodes were 1 mm apart at their closest point. An Erbe VIO300D AC generator was used as a power supply for increased clinical relevance of the experimental setup. Below the beaker was a video camera which faced upward and recorded the progression of the experiment.

The setups of the simulation study and the validation experiment were developed in parallel to ensure that the simulation results be easily comparable with experimental data. In a first attempt, the complete experimental setup was modeled—the instrument’s two jaws and the whole surrounding egg white (see Fig. 2b, upper segment). Simulation results showed that the coagulation process between the electrodes is nearly homogeneous along the *z*-axis (parallel to the instrument jaws). Only a small cutout of the instrument’s central part was modeled for the simulation study (see the lower section of Fig. 2b), thus reducing the necessary number of finite elements to less than 21,000 while still achieving a high resolution with respect to the *x*-*y*-plane.

## Results

### Simulation study

We conducted an in silico study to investigate how the geometry of the electrodes influence the progression of egg white coagulation and the shape of the coagulated egg white, and the thermal spread using our previously published model [19]. Starting from a typical setting with two planar electrodes, namely a wedge angle of 180°, one of the electrodes was changed to a wedge shape with 176°, 160°, 141° and 90°, respectively. Simulations were performed for all five geometries at peak voltages of 35 and $165\,{V_p}$, respectively.

#### Low voltage ($35\,{V_p}$)

**Fig. 3:**
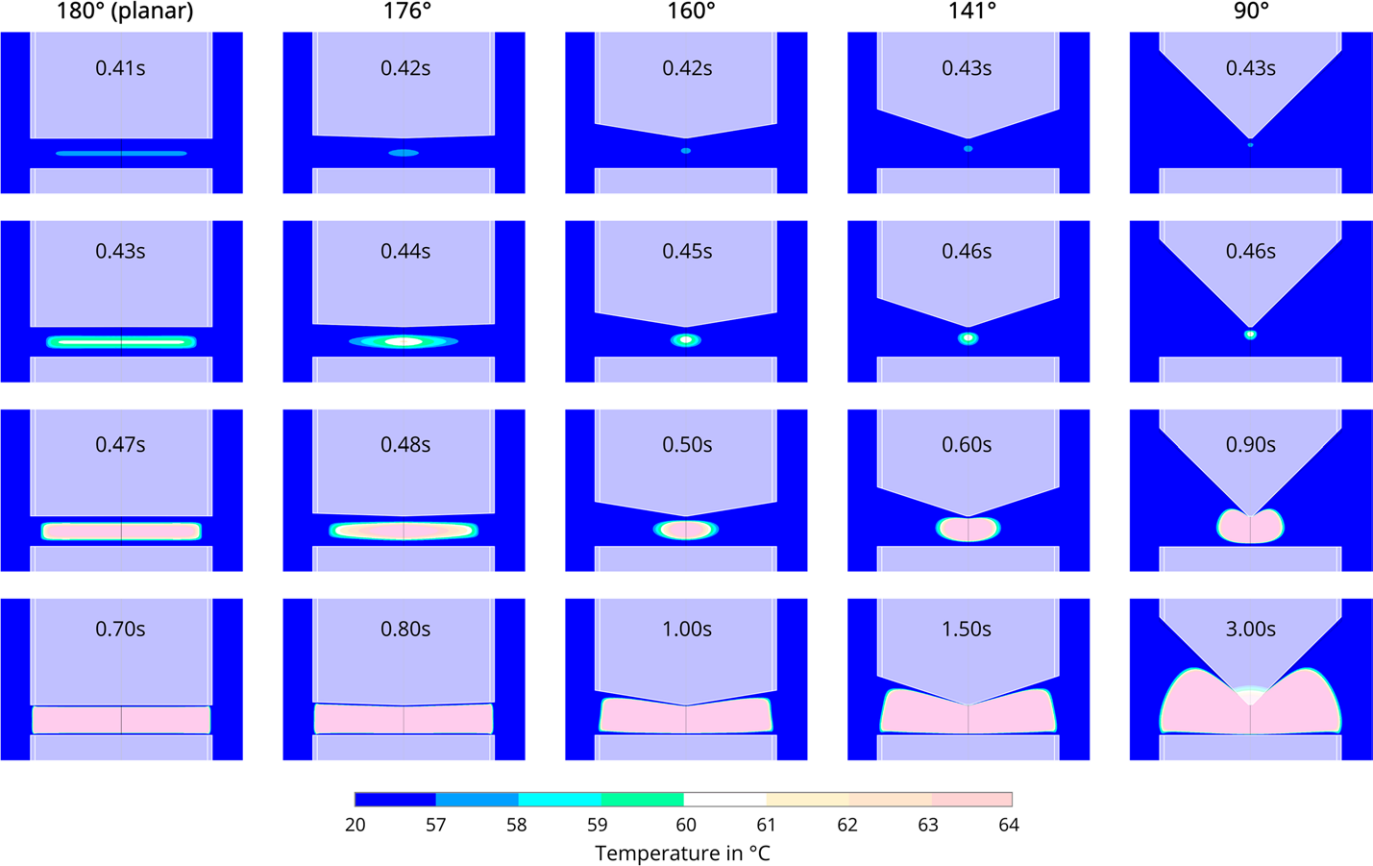
Simulated coagulation progress at $35\,{V_p}$. The *columns* correspond to the wedge angles of 180°, 176°, 160°, 141° and 90° respectively. The *first row* shows the beginning of the coagulation, when temperatures reach locally at least 57 °C. The *second row* shows the moment when temperatures reach locally at least 60 °C. The *third row* shows a representative intermediate step of the coagulation. The *last row* shows the final shape of the coagulated egg white. The instrument’s shape is overlayed in *white*. The *white lines* separate the outer isolator from the electrode

The process of egg white coagulation between the electrodes was simulated for $35\,{V_p}$. Figure 3 shows four representative snapshots for all five geometries. The first row indicates the moment when coagulated egg white is formed for the first time, because the temperature reaches locally at least 57 °C. The second row shows the moment when 60 °C is reached locally. The third row shows a characteristic intermediate step where the shape of the clot is best visible. The last row shows the end of the coagulation process, where most of the egg white between the electrodes is coagulated. The coagulation in the case of two planar electrodes (first column) starts at 0.41 s after the start of the simulation, and it takes only 0.29 s until the egg white is coagulated. The clot is initially formed as a straight line in the center between the two electrodes. From there, the coagulation spreads in a rectangular shape with rounded corners until most of the egg white between the electrodes is coagulated. Only a very thin stripe of egg white, coating the electrode’s surface, remains below 57 °C and is therefore thought to persist in its liquid state.

As the upper electrode obtains an increasingly narrow wedge shape, the coagulation process becomes slower, whereas the initial time point of egg white coagulation is only slightly postponed. Furthermore, the clot shape is significantly affected by the electrode’s geometry. Even the introduction of a slight wedge shape, for instance, altering the wedge angle about just 4°, considerably changes the shape of the coagulated egg white (second column of Fig. 3). Still, the coagulation zone manifests itself between the electrodes, but more in the center as an oval around the symmetry line. The coagulation spreads in an elliptical shape in the early stages. Then it approaches a more rectangular form similar to the planar electrodes with more rounded corners, until almost all of the egg white between the electrodes is coagulated. This occurs at 0.38 s after the formation of the initial clot and at 0.8 s after the start of the simulation.

With decreasing wedge angles, the initially formed clot becomes increasingly punctiform. It shifts towards the tip of the upper electrode. For the electrode with a wedge angle of 160° (third column of Fig. 3), the clot shape remains elliptical during the coagulation process. Even the final shape of the coagulated egg white maintains strongly rounded corners and does not fill all the space between the two electrodes. If the wedge angle is further decreased to 141°, the clot’s intermediate shape changes again and takes on a kidney-like appearance (forth column of Fig. 3), which is even more pronounced for a wedge angle of 90° (fifth column of Fig. 3). The coagulation process in these cases is significantly prolonged and lasts 1.5 and 3 s, respectively. In the case of the steepest wedge shape, this also leads to the heating of the electrode’s tip. The final shape of the coagulated egg white exhibits heavily rounded corners on the side facing the upper electrode, and in both cases does not reach the upper electrode’s surface.

#### High voltage ($165\,{V_p}$)

**Fig. 4:**
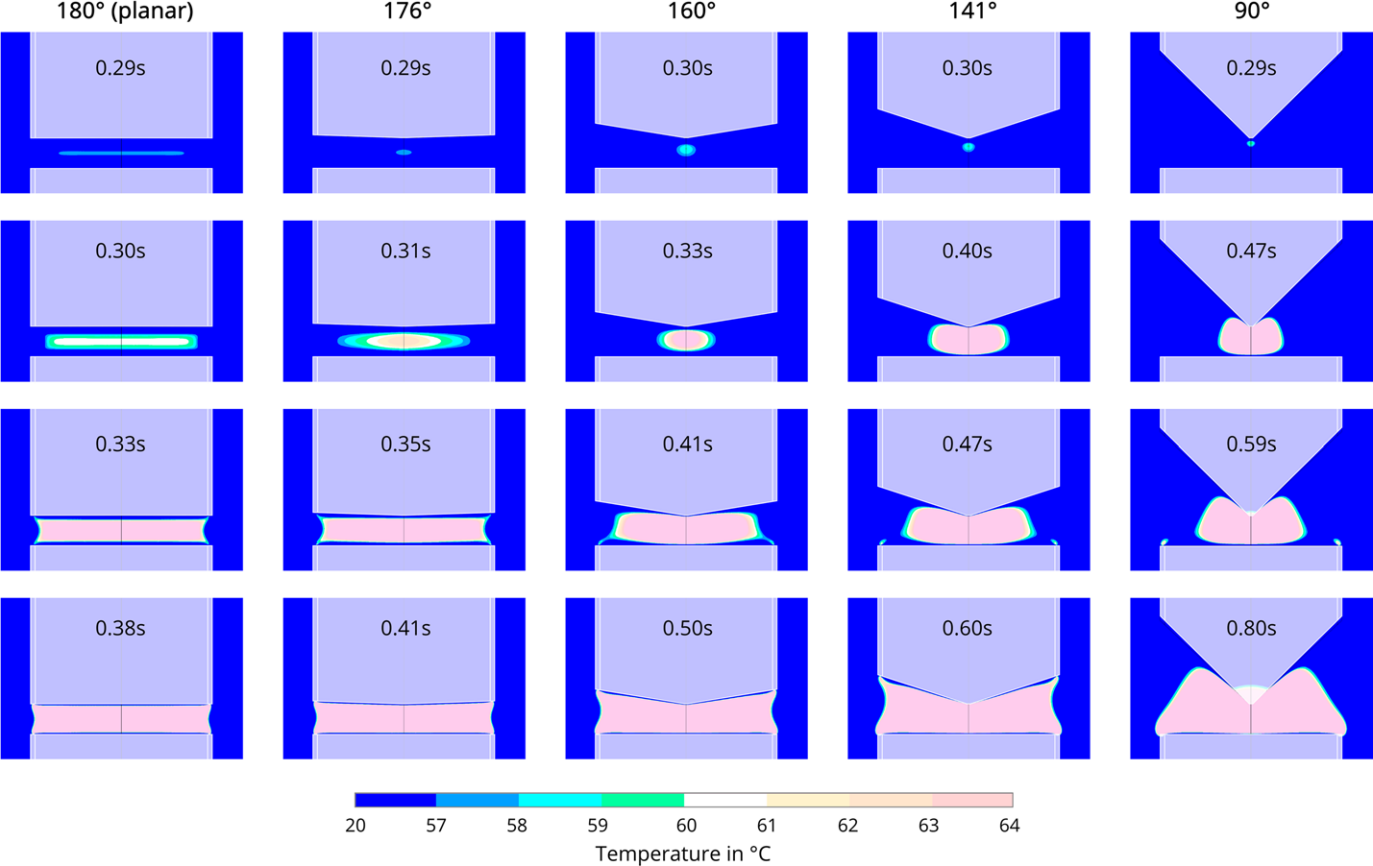
Simulated coagulation progress at $165\,{V_p}$. The *columns* correspond to the wedge angles of 180°, 176°, 160°, 141° and 90° respectively. The *first row* shows the beginning of the coagulation, when temperatures reach locally at least 57 °C. The *second row* shows an early intermediate step where already a significant amount of the egg white between the electrodes is coagulated. The *third row* shows the formation of new coagulation zones at the corner of the planar electrode. The *last row* shows the final shape of the coagulated egg white. The instrument’s shape is overlayed in *white*. The *white lines* separate the outer isolator from the electrode

Figure 4 shows simulations of egg white coagulation using the same electrode geometries but with a peak voltage of $165\,{V_p}$. As in the previous, the first row shows the beginning of the coagulation process when the temperature reaches at least 57 °C locally. Since coagulation progresses quite fast at high voltage, the snapshots where 60 °C are reached locally do not differ significantly from those shown in the first row. The second row therefore highlights an early intermediate step that reflects the characteristic shape of the coagulated egg white. The third row indicates the moment when new coagulation zones are formed at the corners of the planar electrode; the last row once again shows the final shape of the coagulated egg white. The electrode’s geometry has an influence on the shape of the initially coagulated egg white as well as the final clot shape, which is similar to the previously described results at $35\,{V_p}$. Coagulation begins earlier but in the planar case, it also occurs in the middle between the two electrodes. With a decreasing wedge angle, the shape of the initially formed clot reverts to being oval and approaches the tip of the upper electrode. This is similar to the simulations at $35\,{V_p}$. At higher voltages, coagulation progresses faster, in 0.38 s for planar electrodes and in 0.8 s for a 90° wedge angle, instead of 0.7 and 3 s at $35\,{V_p}$ respectively. One notable difference between the simulations with low and high voltages is the occurrence of hotspots at $165\,{V_p}$. These hotspots—newly formed coagulation zones—appear at smaller wedge angles (141° and 90°) at the edges of the planar electrode. They merge with the initially formed central coagulation zone when the coagulation process progresses. Although no hotspots are visible at wide wedge angles, the coagulated egg white distinctly extends towards the edges of the electrodes. Whereas the edges of the fully coagulated egg white at the end of the simulations at $165\,{V_p}$ exhibit a concave shape, the edges at $35\,{V_p}$ possess a convex shape.

### Experimental validation

#### Instrument A

**Fig. 5:**
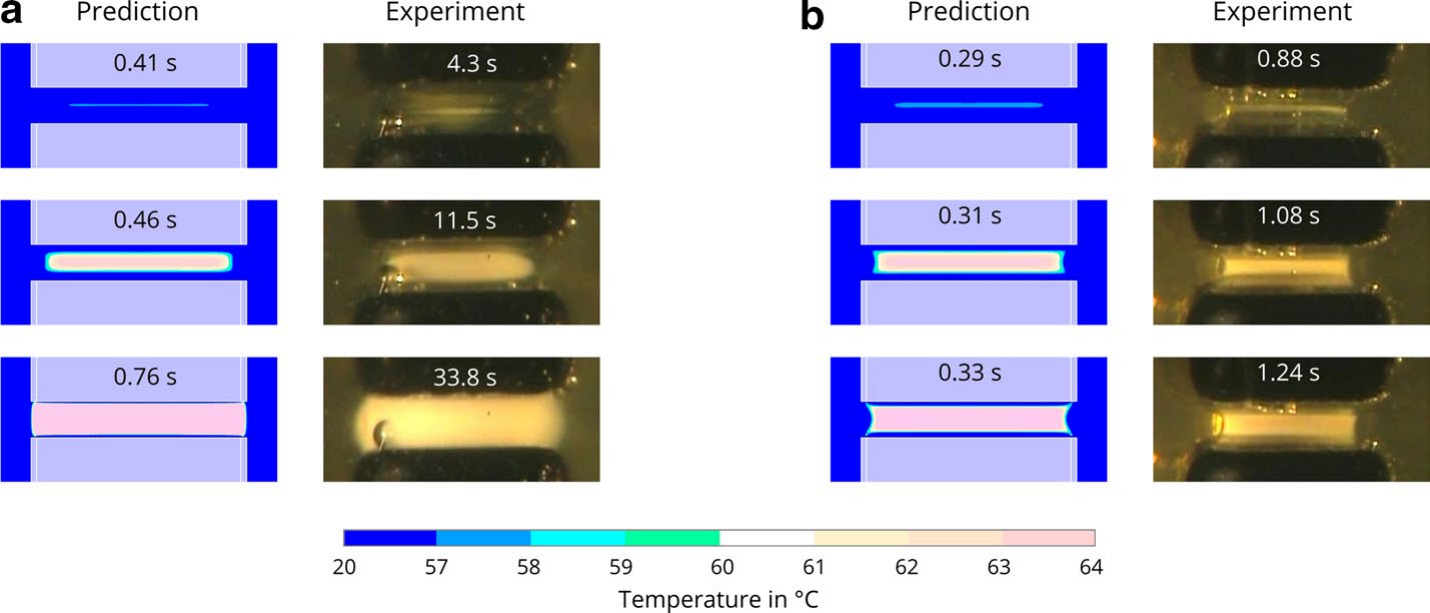
Validation of the predicted coagulation progress for two planar electrodes. Comparison of experimental and simulation results for generator voltages of **a** $35\,{V_p}$, and **b** $165\,{V_p}$, using Instrument A. Provided are representative images of three experiments

Using instrument A, egg white coagulation starts in the middle between the two planar electrodes independent of the applied voltage (see Fig. 5). Depending on voltage, the shape of the coagulated egg white evolves differently over time. At $35\,{V_p}$, coagulation progresses slowly. The cross section of the coagulated egg white resembles a rectangle with rounded corners throughout the whole duration of the coagulation process (see Fig. 5a). The energy input and thus the coagulation speed is considerably higher at $165\,{V_p}$ (see Fig. 5b). The borders of the coagulated egg white appear sharper. The left and right boundaries are slightly bent inwards. After 1.24 s, the explosive formation of bubbles makes further observations of the coagulation progress impossible.

#### Instrument B

**Fig. 6:**
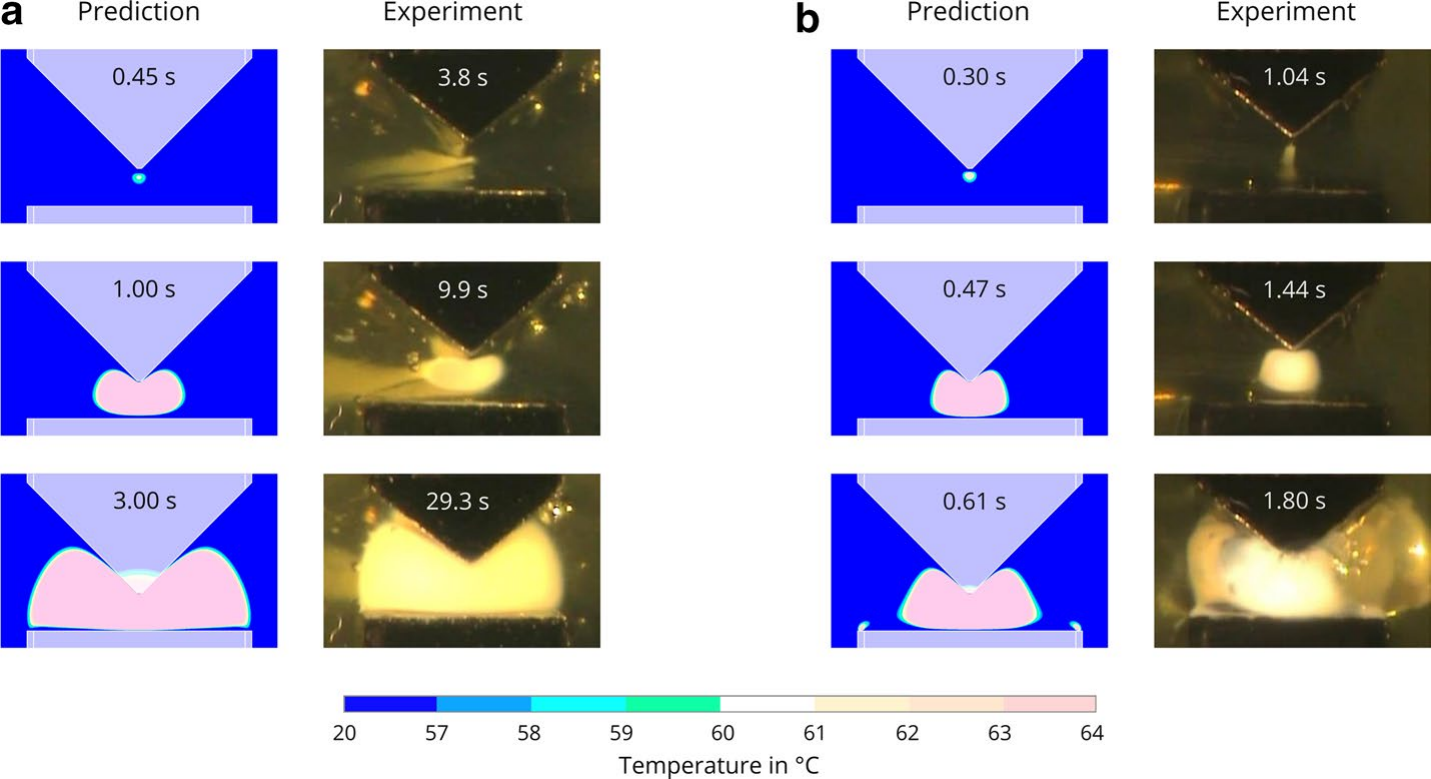
Validation of the predicted coagulation progress for a planar and a wedge shaped electrode. Comparison of experimental and simulation results for generator voltages of **a** $35\,{V_p}$, and **b** $165\,{V_p}$, using Instrument B. Provided are representative images of three experiments

Independent of the applied voltage, coagulation begins close to the tip of the wedge-shaped electrode when using instrument B and grows from there outward (see Fig. 6). At $35\,{V_p}$, the coagulated egg white assumes a characteristic, kidney-shaped cross section in the simulations. At $165\,{V_p}$, the kidney-shape is somewhat jolted and less round. After 1.48 s, the explosive formation of large bubbles interferes with the observability of the further coagulation progress. Nonetheless, hotspots can be observed at the edge of the lower electrode at 1.8 s. A thin layer of egg white coating the planar electrodes remains liquid throughout the coagulation process for both instruments A and B.

## Discussion

### Effect of geometry variations

The simulation study shows a pronounced influence of the electrode’s geometry on the progression of coagulation. An increasingly pronounced wedge-shape results in an increasingly inhomogeneous spatial temperature distribution. Consequently, the initial coagulation zone becomes smaller. It only expands in an increasingly slow manner.

The simulation study illustrates that the geometry of the electrodes is a suitable parameter to selectively influence the temperature distribution. It becomes clear, however, that more complex geometries are required. The implementation and optimization of such geometries can be mostly done in silico and has to be validated by experiments with actual prototypes but only at certain milestones. This work-flow has the potential to significantly accelerate the development process of optimized instrument geometries in a cost-efficient way.

### Validation

The mathematical model captures well the spatial dynamics of the coagulation process. Hotspots, those areas where the coagulation process begins, and the shape of the coagulated egg white are well predicted by the model. The experimental results confirm the distinct influence of both the electrode’s geometry as well as the applied voltage seen in the simulation study. A thin layer of liquid egg white between the coagulated egg white and the planar electrode(s) is seen both in the simulations and the experiments for most of the duration of the coagulation process. The electrodes cool the surrounding egg white and prevent its coagulation. An exception are the edges of the electrodes, where, at high voltages, the high current density causes the egg white to coagulate in those parts.

The temporal dynamics, namely the speed of the coagulation progression, differ significantly from the predicted course, which is also consistent with our previous findings. Actual coagulation commencement at low voltages is approximately ten times slower in the experiments and three to four times slower than predicted by the simulations at high voltages. As already discussed in [19], this large discrepancy cannot be attributed to the modeling approach with the cutout-model since the same behavior was observed when using a model of the full setup. A possible explanation has been to date non-modeled resistances which may prove important when the electrical resistance of the egg white is small due to the close distance of the electrodes. Such resistances might be the contact resistance between the egg white and the electrodes, inductive resistances due to cables, or capacitive resistances caused by the bubbles in the egg white. These resistances are, however, expected to be small. The resulting influence should be far less pronounced.

In our analysis of the experimental results, we found that the AC generator supplied significantly less power than expected. For the validation experiments we used a commercial electrosurgical generator in a voltage controlled mode of operation. It appears, however, that from the point of view of the generator the coagulation of egg white substantially differs from the vessel sealing applications for which it has been designed and optimized. We noticed that the power factor of the generator falls as low as 40% in some experiments with $35\,{V_p}$. This means that at the same voltage level, the actual power output is up to 60% lower compared to the simulations, where an ideal power factor of 100% is assumed. Even at high power settings, the power factor never exceeded 70%. In future validation experiments it is important to choose a power supply that is capable of applying the same energy as in the simulations, e.g. by using a power controlled signal generator. Extending the model to represent the complete electrical setup, including the AC generator, could be another way of significantly improving the quality of predictions with respect to the temporal dynamics.

We also found in video captures of the experiments that there are signs of convective movement. It appears that liquid egg white allows for a significant convective flow of heat. This is another possible reason for the large discrepancy of the temporal dynamics between the simulations and the experiments, particularly at low voltages where the overall progress is slow and heat losses, due to convective heat flows, have all the more influence. The impact of convective heat flow could be investigated in a future work using computational fluid dynamics (CFD) analysis. Additionally, the forceps of the actual instruments are not exactly parallel but open slightly towards the end of the instrument. This means, that there is effectively more egg white between the electrodes in the experiments, resulting in an increased resistance and heat capacity and thus slower heating of the egg white. While the opening angle is small, preliminary studies show that the coagulation speed is indeed reduced if the instrument is modeled more accurately in the simulation.

## Conclusions

In this work, we used our previously developed mathematical model of the egg white coagulation process to evaluate the influence of changes of the electrode geometry on the evolution of the thermal spread. The presented simulation study shows a distinct influence of the geometry on the shape of coagulated egg white. The validation of the simulations revealed that the spatial temperature distribution is properly predicted by the model despite rather large differences regarding the temporal dynamics. The results demonstrate that the proposed in silico evaluation of parameter variations—such as geometry changes—combined with supporting validation experiments is a promising tool for the systematic development and accelerated optimization of vessel sealing devices.

The deviations of the temporal dynamics may be partly explained by non-modeled effects of the coagulation process. There are three aspects of the experimental setup that we think attribute primarily to the observed deviations. First, we used an AC generator that is optimized for vessel sealing applications but supplied reduced power when coagulating egg white. Second, convective heat flows were not considered in the simulation model, but could have had a significant influence on the temperature of the egg white between the electrodes. Third, the jaws of the instrument were not parallel but slightly opened in the simulations, leading to more egg white between the electrodes. Considering these aspects in future works should significantly reduce the deviations of the temporal dynamics between the simulation and experimental results and make the proposed work flow even more advantageous.

## Figures and Tables

**Fig. 7:**
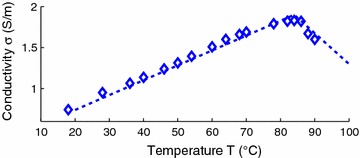
Temperature dependence of the electrical conductivity of chicken egg white. The *blue dotted line* shows the piecewise linear approximation of the measured values. They are indicated by *blue diamonds*

**Fig. 8:**
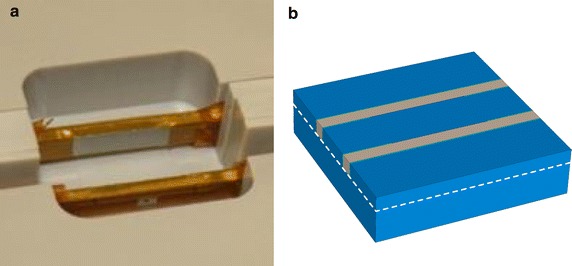
Egg white coagulation with two copper electrodes. **a** Setup of the model verification experiment. **b** Corresponding FE model of the experiment, cut at the symmetry plane. The outer plane is outlined by the *white dashed line*. The electrodes are colored *gray*, the egg white is colored *blue*

**Fig. 9:**
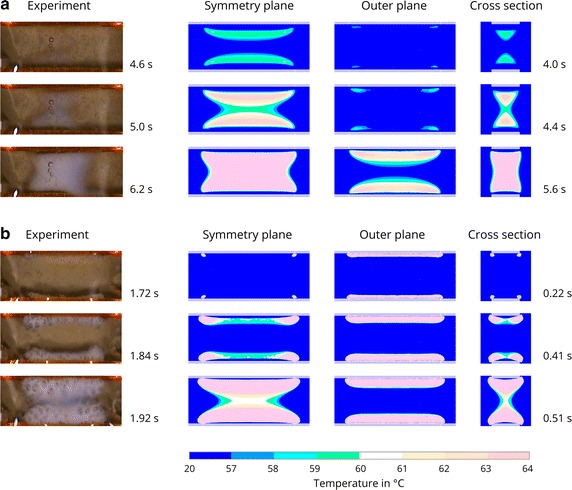
Verification of the mathematical model. Comparison of the experimentally observed progress of the coagulation in the verification experiments and the corresponding FEM simulation results for generator voltages of **a** 50 V_p_ and **b** 200 V_p_. The FEM simulation results show the egg white in between the electrodes. Time values are given from the start of the video capture for the experiment’s results, and from the start of the generator activation for the simulation results. It is assumed that egg white coagulates between 57 and 63 °C in the simulations. Temperatures above 100 °C are displayed in *pink*

## Appendix 1: Model parametrization

In the following, the parametrization of the model will be derived and discussed in detail. A proper parametrization is crucial for obtaining meaningful and reliable predictions.

### Thermal and mechanical properties of egg white

Values reported by Coimbra et al. [20] were used for the thermal and mechanical properties of chicken egg white (see Table 2). Although the thermal properties of chicken egg white show a slight temperature dependence, our investigations suggest that the resulting influence on temperature distribution is negligible. In the following, it is therefore assumed that the thermal properties of egg white are constant.

Evaporation of water is not considered in the model. While this leads to incorrect values of the temperature above 100 °C, these have only a negligible effect on the shape of the coagulated egg white, which is defined by the 60 °C isothermal in the simulations. This was confirmed by simulation studies in which the specific heat capacity was modified to properly account for the vaporization enthalpy.

**Table 2:** Material properties used in the verification simulations

Property	Egg white	Electrode	Isolator
Density $\rho$ (kg/m^3^)	1030	8920	1030
Specific heat capacity $c$ (kJ/kg·K)	3.550	385	2200
Thermal conductivity $k$(W/m·K)	0.55	400	0.12
Electrical conductivity $\rho \; ({\Omega {\text{m}}})$	0.74–1.9	5.56 × 10^7^	1.0 × 10^−12^

### Electrical properties of egg white

Unlike the thermal properties, electrical conductivity shows a strong temperature dependance in the relevant temperature range. It increases by approximately 2%/K [27, 28]. The conductivity also strongly depends on the frequency of the alternating current. Vessel sealing systems typically use frequencies between several hundred kHz and a few MHz, which are outside the conductivity measurements between several dozen MHz and a few GHz as reported in the relevant literature (e.g., [28]). We therefore measured the conductivity of egg white for temperatures between 20 and 90 °C at a frequency of 350 kHz which was used by the vessel sealing system in the experiments later on.

Fresh chicken egg white was slowly heated in a temperature controlled water bath while the electrical conductivity was measured with a Futura PG 13.5 probe (ABER Instruments Ltd, UK). To verify the measurement setup, the conductivity of 0.15 M and 0.05 M NaCl solution was measured. It showed good agreement with the values reported in the literature [29]. The conductivity of chicken egg white increases almost linearly by about 0.0178 S/m K, until it reaches a maximum value of about 1.82 S/m at 84 °C (see Fig. 7). Above 84 °C the conductivity decreases quickly. At the same time, an increasing number of small bubbles is observed in the egg white as the temperature approaches the boiling temperature. Due to the limited amount of measurement points above 84 °C, the decrease of conductivity could not be exactly quantified and we assumed that the conductivity decreases linearly by 0.0375 S/m K for the simulation model.

## Appendix 2: Model verification

In the following, the results of the model verification in [19] are corrected. There, wrong interpolation points were unfortunately used in the look-up table of the electrical conductivity. Consequently, the simulation results shown were computed using differing values of the electrical conductivity that did not exactly correspond to our measurements. While not affecting the general statement of the publication, the error resulted in some deviations of the given temperature distributions which are corrected in the following.

### Experimental setup

The first experimental setup used to verify the mathematical model is shown in Fig. 8a. It consists of two copper electrodes, both with a conductive area of 3 by 10 mm. All non-conducting surfaces were covered with polyimide film. The material properties of the electrode and polyimide film used in this setup are given in Table 2. The two electrodes faced each other at 5 mm apart and were completely covered by the egg white during the experiments. A VIO300D (Erbe Elektromedizin GmbH, Germany) AC generator (350 kHz) set to *Soft Coag* effects E1 and E8, corresponding to peak voltages of 50 and 200 V respectively, was used as the power supply. A video camera mounted directly above the container recorded the progression of the experiment. For the simulation, the central area of the container consisting of the electrodes and the surrounding egg white was modeled, resulting in a 3D FE-model with 69,750 elements (see Fig. 8b).

### Results

The first series of experiments used a setup with a comparably large distance between the electrodes. This reduces the speed of the coagulation process compared to experiments with actual vessel sealing instruments and also improves the observability of the process with respect to space. Using this setup, two distinctly different behaviors of the process can be observed, depending on the energy input. At low energy inputs, the coagulation begins in the horizontal symmetry plane in the vicinity of the electrodes. From there the coagulation progresses inwards, and the area between the electrodes coagulates homogeneously. The coagulation front has soft borders which are probably due to partially coagulated, semi-transparent egg white. This indicates a comparably homogeneous temperature distribution with low gradients. In contrast, the coagulation fronts are much clearer defined at high energy inputs. The coagulation begins at the outer edges of the electrodes. The coagulation zones grow until all egg white in front of both electrodes is coagulated. Only after that does the coagulation continue toward the central area between the electrodes.

Both behaviors are well reproduced by the mathematical model in the FEM simulations. At low energy inputs, the coagulation begins in the symmetry plane near the electrodes. The two coagulation zones soon connect within the symmetry plane, while only small areas of coagulated egg white are formed at the edge of the electrodes. The coagulation zone then extends from the symmetry plane outward until most of the egg white between the electrodes is coagulated. The temperature gradients around the simulated coagulation zone remain small throughout the experiment.

A video camera was used to observe the progression of egg white coagulation as explained in the previous section. Unfortunately, the video capture starts at an unknown short time before the generator was activated and, thus, the absolute times of the video frames and the simulations are incomparable. Nevertheless, we were mainly interested in the spatial evolution of the coagulation process. The offset in absolute times, hence, ought to be considered as a minor issue. Instead, the video frames for comparison with the simulations were chosen as such to represent visually distinguishable steps in the coagulation process. The first video frame was taken when the coagulation of the egg white became explicitly visible for the first time. The second video frame was chosen when the coagulation zone covered the entire electrode surface in the symmetry plane, and the last when the egg white between the electrodes in the symmetry plane was fully coagulated. The simulation times were chosen as such since they represent the same characteristic steps of the coagulation process. Since the coagulation process is very fast at high voltages, the delay between the start of the video capture and the activation of the generator caused a significant offset in the times stated in Fig. 9b. To compare the temporal dynamics of the coagulation process between simulations and experiments, only the relative time spans between the first occurrence of egg white coagulation, the intermediate step, and the final step with fully coagulated egg white have been compared in the discussion.

### Discussion

The verification experiments show that the mathematical model is able to reproduce the typically observed coagulation patterns for both high and low energy inputs. The difference between the experiments and the simulations is small, particularly at low voltages. The time stamps for the experiments given in Fig. 9 are with respect to the beginning of the recording. Unfortunately, there is an unknown offset between the beginning of the recording and the activation of the generator. This is not a big issue at low voltages, where the coagulation progresses only slowly, but the offset becomes all the more relevant at high voltages. To verify the temporal dynamics, it is more meaningful to compare the relative times between the captured images and the relative times between the corresponding simulation snapshots. At low voltages, the relative times of the simulations closely match the experimentally observed times. At high voltages, the relative times differ slightly from the corresponding experimentally observed times. The difference is acceptably small, particularly when considering the strong occurrence of non-modeled effects like bubble formation.
